# ADVANCING RURAL GERIATRIC SERVICES: LESSONS FROM THE VETERANS HEALTH ADMINISTRATION

**DOI:** 10.1093/geroni/igae098.0977

**Published:** 2024-12-31

**Authors:** Emily Franzosa, William Hung, Bret Hicken

**Affiliations:** Chair; Co-Chair; Discussant

## Abstract

Almost 3 million rural Veterans receive care from the Veterans Health Administration (VHA) and more than half are 65 and older. But, rural older Veterans may have limited access to specialized, interdisciplinary geriatric care services. VHA’s nationwide system is tasked with supporting Veterans wherever they live, a mission that comes with unique access challenges for Veterans far from VA hospitals or clinics. In a system responsible for managing many priorities and needs, prioritizing rural geriatrics services can be challenging. Our symposium highlights efforts to develop successful strategies for expanding rural geriatrics nationally. First, Dadgostar et al. share results from a project to understand geriatric service needs across VA, with the goal of leveraging data to map areas that could benefit from service expansions. Next, Kelley and colleagues discuss findings from interviews with VHA leaders on how priorities are determined at the local, regional and national level, and how rural geriatrics services fit with leaders’ visions. Franzosa et al. share a rural geriatrics “advocacy field guide” to support geriatrics champions in the field to gain support for local resources. Finally, Dryden et al. share an example from a successful rural geriatrics program, GRECC Connect, and discuss how these implementation lessons can inform the development and scale of new programs. Through the lens of rural geriatrics, this symposium highlights effective strategies for health care leaders to move their priorities ahead within their own systems.

